# Perceptual oddities: assessing the relationship between film editing and prediction processes

**DOI:** 10.1098/rstb.2022.0426

**Published:** 2024-01-29

**Authors:** Alice Drew, Salvador Soto-Faraco

**Affiliations:** ^1^ Multisensory Research Group, Centre for Brain and Cognition, Universitat Pompeu Fabra, Carrer de Ramon Trias Fargas, 25-27, 08005 Barcelona, Spain; ^2^ Institució Catalana de Recerca i Estudis Avançats (ICREA), 08010 Barcelona, Spain

**Keywords:** cognitive conflict, predictive processing, theta oscillations, film editing, electroencephalography, neurocinematics

## Abstract

During film viewing, humans parse sequences of individual shots into larger narrative structures, often weaving transitions at edit points into an apparently seamless and continuous flow. Editing helps filmmakers manipulate visual transitions to induce feelings of fluency/disfluency, tension/relief, curiosity, expectation and several emotional responses. We propose that the perceptual dynamics induced by film editing can be captured by a predictive processing (PP) framework. We hypothesise that visual discontinuities at edit points produce discrepancies between anticipated and actual sensory input, leading to prediction error. Further, we propose that the magnitude of prediction error depends on the predictability of each shot within the narrative flow, and lay out an account based on conflict monitoring. We test this hypothesis in two empirical studies measuring electroencephalography (EEG) during passive viewing of film excerpts, as well as behavioural responses during an active edit detection task. We report the neural and behavioural modulations at editing boundaries across three levels of narrative depth, showing greater modulations for edits spanning less predictable, deeper narrative transitions. Overall, our contribution lays the groundwork for understanding film editing from a PP perspective.

This article is part of the theme issue ‘Art, aesthetics and predictive processing: theoretical and empirical perspectivess’.

## 1. Introduction

‘You're seeing it all in your mind's eye, you're inferring it. And this is the fourth aspect of cinema that's so special. That inference. […] you take one shot, you put it together with another shot, and you experience a third image in your mind's eye that doesn't really exist in those two other images. […]—if you change the timing of the cut even slightly […], then that third image in your mind's eye changes too. And that has been called, appropriately, I believe, film language.’ – Martin Scorsese [1].

### (a) Cinema and everyday vision: an apparent paradox

In his interview for the *New York Review of Books* [1], Martin Scorsese could not have put it any clearer: filmmakers are no strangers to the importance of inference-making during film viewing. Cinema poses an apparent paradox of vision: in films, events differing in time, space, actions, characters or situations are spliced, forming sequences of sensory input that are radically different from the visual flow of our everyday life experience, where visual input is continuous, or at least predictable from self-generated actions such as saccades. With a present-day average shot length of under 4 s for English-language films [2], one would think that such contrived changes at such a high rate should pose a tremendous challenge to our perception. However, this is far from the case: when watching films, we parse visual transitions mostly seamlessly, piece together individual shots into a coherent narrative structure, and segment different parts of a story into its component episodes. To this end, most cuts in film are designed to be perceptually inconspicuous and largely succeed in being so [3,4]. In fact, film viewing is considered by most to be a relaxing form of entertainment, not a visual puzzle.

Film theorists have argued that cuts largely go unnoticed since they mimic visual interruptions that occur naturally during blinks and saccades [3,5], although there is currently no evidence that these co-occur [6]. Films do not appear perceptually unnatural to us thanks to carefully designed editing techniques. Narrative coherence is partly induced by montage—the editing and stringing together of different shots [4,7,8] to create continuity or discontinuity around editing boundaries and engender feelings of fluency/disfluency and tension/relief, to name a few. However, for these techniques to succeed, perceptual processing must work toward filling in gaps and smoothing over blatant breaches in sensory continuity. Not only that, but unlike everyday life perceptual inferences, those we generate when watching films are in keeping with an overarching narrative that can supersede expectations based on prior understanding of the behaviour of people and objects, such as for instance the laws of Newtonian physics [9]. As such, film viewers often bridge abrupt changes in visual input across time and space at the service of narrative, although such spans would be disconcerting, if not physically impossible, in real life. In what follows, we argue that our ability to draw inferences about the relationship between images across film cuts is essential to narrative sense-making [4] and posit that the success of editing techniques is heavily reliant on predictive processes in the brain.

### (b) Predictive processing, conflict monitoring and aesthetic pleasure

Outside of film viewing, it has been suggested that our subjective experience of vision as a continuous flow is an elaborate illusion made possible by a combination of perceptual mechanisms [10,11]. According to several current theories, perception can be cast as a continuous process of prediction [12,13]. One of such theories, called predictive processing (henceforth, PP) holds that the brain tries to predict upcoming sensory input drawing on an internal model built from prior experience as well as the currently available sensory context [13]. The disparity between these top-down predictions and the actual bottom-up sensory information (e.g. unanticipated changes in the environment) generates prediction errors (mismatch responses) which, in turn, are propagated up the system for further model adjustment. The loop between prediction, comparison and adjustment in the goal of minimising error in the long-term leads to constant fine-tuning and adaptation of internal models [14,15]. Importantly for our purposes, according to PP, prediction errors have stronger or weaker effects depending on their ‘weight’: only the most conspicuous and the ones that are considered particularly salient (i.e. high in ‘precision’) trigger attention orienting to alert the agent to something unusual in the environment, while most are not considered notable and are consequently resolved before even reaching awareness.

PP in this respect joins another prominent theory of cognition: conflict monitoring and cognitive control, or conflict monitoring theory (CMT) [16–19]. Despite sharing numerous similarities, a clear account of these similarities is surprisingly lacking in the literature (though see [20] for an exception). CMT refers to a set of functions by which the brain monitors and flexibly adjusts to changes in the environment. The theory proposes that fronto-medial (fm) brain structures such as the anterior cingulate cortex (ACC), are responsible for the monitoring and detection of conflicts during information processing: if incompatible representations are simultaneously activated, then adjustments are triggered, putatively invoking areas in the dorso-lateral prefrontal cortex (dlPFC) to recruit the relevant resources to adapt and respond to the conflict, minimising any consequent disruption and future reoccurrence. This conflict system ensures efficient use of resources. A lot of the empirical evidence for conflict processes comes from classic sensorimotor protocols inducing incompatible response tendencies (e.g. Stroop or Flanker tasks).^1^ However, the original formulation of the framework, as well as later updates make clear that conflict, like prediction errors in the PP framework, can occur at any level of the information processing hierarchy, including perceptual representations [16,17,22,23], a claim that is corroborated by a mounting body of evidence across different domains (semantics [24,25], crossmodal and visual perception [26–29], and surrealism in art and advertisement [30]). Should the theory of conflict monitoring be applicable to sensory processing and perception at large, then it could serve as the regulatory mechanism for prediction error. We elaborate on the possible links between these two frameworks in greater detail in the Discussion (§4c).

Of course, cinema, as a form of entertainment, constitutes a special case of perception—one, as we noticed, potentially much more riddled with uncertainty and mismatches between our predictions and the actual sensory inputs. Precisely for this reason, it provides an ideal terrain for theories of PP that place uncertainty and prediction error minimisation at the heart of human information processing. Several recent PP accounts emphasise the importance of affect and learning in understanding the human drive to engage with the arts, highlighting the importance of ‘error dynamics’ over time (the long-term minimisation of uncertainty or prediction error) [31–35]. Put simply, the reduction of prediction errors over time induces positive affect, particularly if it exceeds our expectations, while the increase in prediction errors over time induces negative affect, again, particularly if it exceeds our expectations. Crucial to this account is the ability to *resolve* the errors (i.e. learning): for this reason it is important that errors fall within an optimum that allows resolution, a ‘sweet-spot’ often illustrated as the apex of an inverted U-curve relating complexity and affect [35–38]. Art, for instance visual arts [39], music [40] or cinema [41] are thought to represent an opportunity to engage in such dynamics, offering just the right amount of challenge to allow for resolution. Understanding error dynamics helps to explain why positive affect often follows the resolution of conflict (unexpected reward) both in simple low-level tasks (such as Stroop) and more complex scenarios such as music enjoyment [38,42] and why unknown environments can lead to greater reward (despite higher uncertainty) than more certain situations [18,43], in line with theories of curiosity [44,45] and of epistemic foraging [46] which, interestingly for the current argument, have also been linked to ACC function [47,48]. In sum, states of uncertainty^2^ are often actively sought out *because* they provide us with opportunities to resolve challenges (and subsequently gain information), with art and aesthetics being a prime example of this.

### (c) Neural correlates of error signals

While the theoretical grounding of PP is well fleshed out [49,50], its neurophysiological correlates are much less well established (see [51,52] for reviews on this subject). Prediction error and surprise signals have long been suggested to be encoded by the ACC [53–57] as well as predicting reward from uncertain and volatile environments [53,58,59] and error likelihood [55]. Midbrain dopamine neurons also reportedly encode error signals and reward value predictions [60], with the ACC projecting to the ventral tegmental area, an important area for dopaminergic prediction errors [61]. Furthermore, error signals are increasingly thought to overlap with the neural correlates of conflict monitoring and detection in the CMT, which are far more empirically grounded: early and more recent models of ACC function explicitly bring together conflict and prediction error signals, as a unifying trend towards a single computational framework able to account for these as well as a variety of other signals ([53,58,62]; see [63] for a recent review). The ACC is also reported to represent perceptual predictions in the context of event segmentation [64]. In electroencephalography (EEG), an increase in oscillatory power in the theta band (5–7 Hz) in mid-frontal areas distributed over the ACC, has been proposed as the *lingua franca* reflecting a common adaptation mechanism in a variety of situations involving ambiguity (prediction errors, input conflict, post-error adjustments, surprise and novelty) [65–70]. This oscillatory correlate has since been widely employed in conflict tasks at varying levels of information processing [27–30,71], and we adopt it in what follows as a neural signature of prediction errors between anticipated and actual sensory input at edit boundaries in cinema.

### (d) Cinema: a useful case study for the predictive processing framework

Back to film, cinema is a useful case study for PP since it purposefully breaches sensory expectations and induces uncertainty to foster engagement and suspense. Montage, according to continuity editing theory, relies on and exploits human perceptual processes in at least three important ways. First, it encourages viewers to make predictions about future events across shots. Secondly and thirdly, filmmakers rely on backward and forward causal reasoning [72,73]. When directed backwards, viewers must piece together different shots into a coherent sequence, filling in gaps in information. When directed towards the future, viewers are invited to guess the relevance of new information, seemingly unrelated to prior sensory information. Crucially, while montage encourages the generation of prediction, it does not ensure that these predictions will be met. Early Russian films using the Soviet montage theory championed the power of montage to ‘constrain inference processes’ through backwards oriented inferences [4,72], in other words to play with the viewers’ ability to infer and interpolate missing parts of the narrative from a sequence of shots: the greater the discontinuity between shots (violation of prediction), the greater the need for inference (hence, greater imaginative liberty for the ‘image in the mind's eye’) on the part of viewers, epitomised by the ‘Kuleshov effect’ [74,75].

### (e) Overview of previous studies

Cinema and narrative are relatively underexplored artforms from a PP perspective, but two studies do make explicit mention of the framework, which they apply to horror [41] and ‘cognitively challenging films’ [73] in particular. In both studies, the authors claim that the cinematic propensity to maximise rather than minimise prediction errors allows us to explore volatile scenarios that teach us to embrace uncertainty by expanding our ‘cognitive-perceptual repertoire’ (i.e. being exposed to new situations—fittingly, the ability to interpret cinema improves with experience [76]) [11].

Another study, by Magliano *et al*., shows that the number of predictive inferences in film depends on the presence, type and amount of various cinematic devices, among others, *mise-en-scène*, sound, and most frequently, montage [72]. Furthermore, edits made according to continuity editing rules result in greater edit blindness (i.e. missed edits in an explicit edit detection task) than those not in adherence to them [6], with the proportion of missed edits as well as participants' response times varying significantly between edit types. In a similar vein, Levin *et al*. observed that participants’ assessment of continuity at editing boundaries was not affected by introducing 400 ms overlaps or ellipses between shots, unless they were explicitly instructed to scrutinise the edits [77]. Magliano & Zacks [78] found that the processing of editing boundaries is reflected differently, both in behaviour and in BOLD responses measured using functional magnetic resonance imaging (fMRI), according to three levels of narrative depth [4]: continuity edits (which contain transitions between shots but are continuous in space, time and action—i.e. a shift in camera position), spatial-temporal discontinuities (discontinuous in space and/or time but continuous with the main action in the scene), or action discontinuities (discontinuous in action as well as space or time). Behaviourally, action discontinuities were most frequently associated with the beginning of new meaningful events. In the fMRI, spatial-temporal discontinuities and continuity edits showed BOLD responses to changes of location and changes in stimulus input respectively, as well as the former showing patterns of activation linked to attention-driven downregulation across these visual boundaries suggesting the activation of selective neural processing to stifle low-level feature discontinuities at the service of narrative. Eye movements following edits have also been shown to increase proportionally to the distance across cuts between important objects in the scene [79]. Finally, multiple EEG studies report a decrease of oscillatory power in the alpha band (8–12 Hz) following shot changes while watching television, a pattern commonly associated with attentional allocation and explainable as an internal orienting response to the novel information detected [80,81].

While to our knowledge, no study to date has addressed film editing from a PP perspective, collectively, the findings above suggest that brain responses following editing boundaries might reflect the degree of sensory input unaccounted for by perceptual predictions, that is prediction error, validating filmmakers' efforts to either draw viewers’ attention to editing boundaries or conversely make these inconspicuous. According to this account, the magnitude of the error signal should be sensitive to the different narrative levels of montage theory (cf. §2a(vi)).

### (f) Scope of the present study

The two experiments reported in this study test the proposal that breaches in low-level visual continuity (sensory transients at edit points) produce prediction errors, corresponding to the relative unpredictability of the visual change in low-level sensory input. We expect neural mechanisms of error signalling to be selectively engaged to bridge these low-level discontinuities at the service of narrative. In addition, we anticipate that the magnitude of these signals at edits should depend on the predictability of the visual transition at the narrative level. In Experiment 1, we measured the neural correlates of error/conflict signals, i.e. fm-theta band activity in the EEG, while viewers were asked to passively watch fragments of movies. We hypothesise that prediction errors will be reflected as an increase in fm-theta power following action discontinuity edits, relative to space–time and continuity edits. We also recorded electrodermal activity for exploratory purposes (analysis available in the electronic supplementary material). In Experiment 2, we measured performance of a new group of participants as they actively detected edit points in the same movie fragments. We anticipated that the least noticeable events according to our hypothesis (here, continuity edits and spatial–temporal discontinuities) should produce slower response-times and should be less noticeable than action discontinuities, since the former span only small gaps in the narrative. Both experimental designs and analysis pipelines were pre-registered on the Open Science Framework at https://osf.io/hvyfm (Experiment 1—pre-registered during data collection but prior to data analysis) and https://osf.io/s8nwq (Experiment 2—pre-registered after data collection but prior to data analysis).

## 2. Experiment 1: passive viewing electroencephalography

### (a) Methods

#### (i) Participants and sample size estimation

Thirty healthy subjects (mean age: 25.1 years old; 15 female, 15 male) participated in the experiment and were recruited through the Centre for Brain and Cognition of the Universitat Pompeu Fabra participant database. Inclusion criteria comprised healthy vision and not being under medication. All received 10€/hour in return for their participation, provided written informed consent in advance, and were naive to the purpose of the experiment. This study was approved by the CIEC ethics board of Parc de Salut Mar (Universitat Pompeu Fabra).

The sample size was calculated using G*Power [82]. Given our practical affordances and aiming for a statistical power of >0.80 and an effect size of *d* = 0.45 [83], the sample (N) returned by G*Power was of 32. Additionally, using equivalence testing on R, we were able to determine the minimal effect size we could reliably detect with the sample that we could afford (for a sample of 30 participants with 80% power, the minimal effect size of interest must be outside [−0.53,0.53]). Taken together, we decided to collect a sample of 30 participants.

#### (ii) Stimuli and task

The experiment was built using Psychopy's experiment builder, (version 2020.2.10, [84]). Participants passively viewed seven continuous clips (total duration 19.54 min, mean 2.79 min, s.d. = 0.69 min) from the following chosen feature films: *The Good, the Bad, and the Ugly* (Sergio Leone, 1966; training clip), *La Grande Belleza* (Paolo Sorrentino, 2013; two clips), *Le Ballon Rouge* (Albert Lamorisse, 1956), *Le Quattro Volte* (Michelangelo Frammartino, 2010), *Somewhere* (Sofia Coppola, 2010), *Laurence Anyways* (Xavier Dolan, 2012), *Bin-jip* (Kim Ki-duk, 2004).

These clips were selected based on whether they contained edits of the three types described below (cf. §2a(vi)) and contained no dialogue,^3^ to avoid interference from linguistic processes. Film clips were in the XviD MPEG-4 digital video format at a resolution of 720 × 576 and 25 fps (DVD quality). The experiment was designed on a window size of 1920, 1080 pixels with the clips centred on an HP Omen 25 screen with of a width of 960 and a height of 540 (units relative to the window size) overlaid on a grey background (RGB values from −1 to 1 [0,0,0]). Each clip was presented once and in the same order for each participant.

#### (iii) Procedure

**Figure 1.**
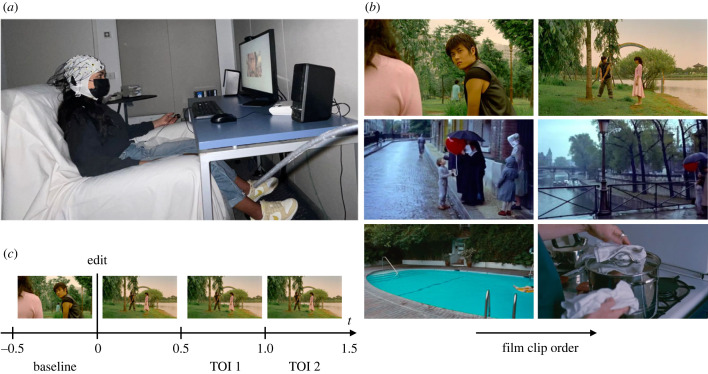
(*a*) An image of the experimental setup: a participant performs Experiment 1. (*b*) Illustrative examples of the three types of edits analysed in the study (top to bottom), displaying the pre-edit and post-edit frames (left to right). Continuity edit: the only change is a change of camera angle, all other main features remaining constant. Spatio-temporal discontinuity: a spatial and temporal change within a same scene identified by continuity of action (the boy walks through the city). Action discontinuity edits: a man is floating on a lilo, the next shot features a man cooking in a kitchen. (*c*) Trial timeline depicting the baseline period (500 ms pre-edit), edit and both time windows of interest for analysis.

Participants were seated in a dimly lit and sound-attenuated room (acoustic insulation approximating 55 dB). Clips were displayed on the screen placed at approximately 90 cm from participants' heads, with the soundtrack audible through Creative T-20 loudspeakers (sound adjustable to a comfortable level, average range of 32–42 dB[A]). Once the EEG and electrodermal activity (EDA) setups had been mounted, participants were instructed to simply watch the film clips passively, while trying to minimise blinks (to avoid contamination in the EEG signal). One training clip (excerpt from *The Good, the Bad and the Ugly* of 1 min 45 s duration) was played with experimenters still present. As in the task stimuli, the training clip was followed by two ‘yes/no’ content questions in English about the fragment, serving to assess whether participants were paying attention to the clips (participants were informed they could contact the experimenter if they did not understand the questions). For example, the content questions included: *Is the boy's balloon always the same colour?* (*Le Ballon Rouge*) or *The clip features a cow on a table* (*Le Quattro Volte*)—the complete list of questions can be found in the electronic supplementary material. Once the training had finished, the experimenter left the room and the task began. At the end of the experiment, participants were asked whether they had previously seen any of the films (see electronic supplementary material). The experimental setup can be seen in figure 1*a*.

#### (iv) Electroencephalography recording and preprocessing

During the experiment, EEG data were acquired using a 64-electrode system (actiCAP, Brain Products GmbH, Munich, Germany) placed in accordance with the 10–10 international system. The ground electrode was placed on AFz and the online reference on the right mastoid. Electrodes for offline re-reference were placed on right and left mastoids. The vertical electrooculogram was recorded by an electrode underneath the right eye, and the horizontal electrooculogram at the outer canthus of the right eye. Impedance was kept below 10 kΩ for all electrodes. Signal was recorded via BrainVision Recorder (Brain Products GmbH, Munich, Germany) at a sampling rate of 500 Hz, connected to stimulus presentation via parallel port.

Preprocessing and analyses of EEG data were done using Fieldtrip [85] and custom-made code in Matlab (please refer to the data accessibility statement at the end of this manuscript for a link to scripts and datasets). The continuous EEG data were segmented into trials surrounding continuity edits; these were defined as segments from −0.5 s prior to and 1.5 s following the edit moment (*t* = 0, by convention). The window −0.5 s to 0 was used as baseline. Then, we selected two 500 ms time windows of interest after the edit, a first time window from 0.5 s to 1 s, and a second time window from 1 s to 1.5 s (the trial timeline can be seen in figure 1*c*). The window length was selected to include at least 3 cycles of the central frequency of interest (6 Hz) in the theta band. Given this required trial length, the minimum shot length for analysis was of 2 s to avoid overlap between trials (shorter shots, *N* = 7, were not considered). Both time windows were initially chosen since we did not know the exact latency of the possible effect: while fm-theta adjustments can be reflected in close vicinity to the stimulus (and until 750 ms post-stimulus), this has typically been tested on low-level stimuli, hence the inclusion of a second later time window given the high-level nature of narrative stimuli. Furthermore, cuts are often followed by a large event-related potential (ERP) [86] related to the visual transient, hence our decision to observe theta responses once this ERP could no longer be a confound. Independent component analysis was used to reject EEG components corresponding to blinks, heartbeat or eye movements. Sixty-four components were obtained from the decomposition (with the ‘runica’ method in Fieldtrip; mean = 2.27 components discarded per participant, almost systematically including components for blinks and lateral eye movements, upon visual inspection). Data were then further visually inspected to manually reject segments contaminated by other identifiable motor related artefacts (mean = 1.03% of trials discarded per participant). Bearing in mind our pre-registered minimum of 30 trials per edit type, one participant was discarded based on this criterion from the pre-registered analysis but included in exploratory analyses.

#### (v) Exclusion criteria

Participants with fewer than 70% correct answers to content questions were to be discarded from further analysis (*p* = 0.09 threshold determined by simulating 100 000 random answers to 14 yes/no questions). No participant was discarded according to this criterion; response rates for each clip can be found in the electronic supplementary material.

#### (vi) Edit type coding

A detailed account of our editing coding can be found in the electronic supplementary material. We based our taxonomy on that used by Magliano & Zacks [78], due to its consideration of various levels of narrative depth, first identifying all editing boundaries (cuts, fade-ins, fade-outs) for all clips before marking each editing boundary as one of three types: continuity edit, spatial-temporal discontinuity and action discontinuity. Continuity edits (*N* = 48 edits) contain transitions between shots, but are continuous in space, time and action (i.e. a shift in camera position). Spatial-temporal discontinuity edits (*N* = 31 edits) are discontinuous in space and/or time but continuous with the main action in the scene. Action discontinuity edits (*N* = 31 edits) are discontinuous in action as well as space or time. Examples of each edit type can be seen in figure 1*b*.

#### (vii) Power analysis

According to the pre-registered hypotheses (https://osf.io/hvyfm/), the analysis was focused on the theta frequency band (5–7 Hz) at fronto-central electrode sites. We used a Fast Fourier transform (with a Hanning taper zero padded up to 1 s) to extract the power in the frequency bands and time windows of interest. We measured the power in the pre-selected time windows expressed in decibels (dB), always baseline-corrected (by subtracting the average power in the baseline window). While activity was measured over the whole scalp, a region of interest (ROI) was pre-defined for the fronto-medial region (Fz, FCz, Cz). This ROI was based on previous studies of conflict processing [68].

Two analyses were pre-registered and performed for each time window of interest (0.5 s–1 s and 1 s–1.5 s post-edit). First, we estimated theta power and tested if it increased (with respect to baseline) following the edit for all edit types combined. Second, we compared post-edit theta power (again, baseline corrected) across the different edit-types. Power contrasts were calculated for each frequency and electrode of interest and, subsequently averaged for comparison across edit types.

#### (viii) Source localisation

An exploratory source localisation was performed to verify that the theta band increase observed in the power analysis was consistent with the fronto-medial brain areas specified in our hypothesis. Source localisation was performed using Dynamic Imaging of Coherent Sources (DICS) [87]: a spatial filter operating in the frequency domain applied to the data in every voxel of a pre-computed standard grid over the brain, amplifying signal from each location and attenuating that in others. We calculated the leadfield using a standard boundary element method template available in Fieldtrip (version 20210507) and set our grid spacing to 1 cm. For each participant, power and cross-spectral density was calculated centred on the effect found in the sensor level analysis (theta frequency band (5–7 Hz), for all edit types combined) using the same parameters as in the sensor-level analysis. The common filter across baseline (−0.5 s–0 s before edits) and time window of interest (0.5 s–1 s following edits) was calculated for each participant using a regularisation parameter of lambda 5%. Monte Carlo cluster correction was used to correct for multiple comparisons across voxels for the power contrast across baseline and time window of interest in dB (alpha threshold = 0.05, number of iterations = 10 000) [88].

#### (ix) Time course of theta activity at mid-frontal electrodes

An exploratory analysis was performed to examine the time course of theta activity surrounding edits throughout the pre-registered trial length (−0.5 s–1.5 s). We calculated oscillatory power for each participant and edit type using 500 ms sliding windows in steps of 20 ms and 1 Hz in the corresponding ROI for the theta band (5–7 Hz) using a short-time Fourier transform. A Hanning taper was applied to reduce spectral leakage. Power was baseline-corrected by subtracting the average power of the baseline period from the power following edits in dB. Data was then averaged over frequency, electrode and trial to obtain a single time-series per participant and edit type. Grand averages were then obtained across all participants for each edit type. We performed a Monte Carlo cluster correction for multiple comparisons [88] (alpha threshold = 0.05, minimum neighbour channels = 0, number of iterations = 10,000, cluster selection based on maximum size).

#### (x) Extended time-frequency analysis at mid-frontal electrodes

A wider range of frequencies (2–30 Hz in steps of 1 Hz) for the ROI electrodes were analysed, to produce a time-frequency map for all edits combined to check if other frequencies were involved in the effect.

#### (xi) Time course of parieto-occipital alpha oscillations and time frequency representation at parieto-occipital electrode sites

Alpha suppression (a decrease of oscillatory power in the alpha band, 8–12 Hz) has been reported following cuts [80] and attributed to an increase in attentional engagement directed towards the newly presented information. We ran an exploratory analysis to address the time course of alpha activity using the same parameters as for the theta band but for the alpha frequency range (8–12 Hz) and at parieto-occipital electrode sites (P7, P8, PO7, PO3, POz, PO4, PO8, O1, Oz, O2). Generally, decreases in alpha are purportedly reflective of attentional adjustments via functional inhibition of irrelevant information across the sensory cortices [89–92]. We then explored the time-frequency representation over the pre-registered trial length regarding activity at the electrode sites of the same selection of parieto-occipital electrodes.

#### (xii) Electrodermal activity

As a further exploratory analysis, electrodermal activity (EDA) was measured during the task. Details and results of this analysis can be found in the electronic supplementary material.

### (b) Results

#### (i) Power analysis: induced theta power after edits

We tested whether fm-theta power (in dB) increased with respect to baseline (0.5 s window prior to the edit) for each time window of interest (0.5 s–1 s and 1 s–1.5 s) after the edit, for all edits combined. All contrasts were one-tailed (as per the directional hypothesis), with *α*-level = 0.05. The results showed a significant increase in theta power following edits in the first time window (*t*_28_ = 2.095, *p* = 0.02; Cohen's *d* = 0.39), but not in the second (*t*_28_ = 0.821, *p* = 0.21).

**Figure 2.**
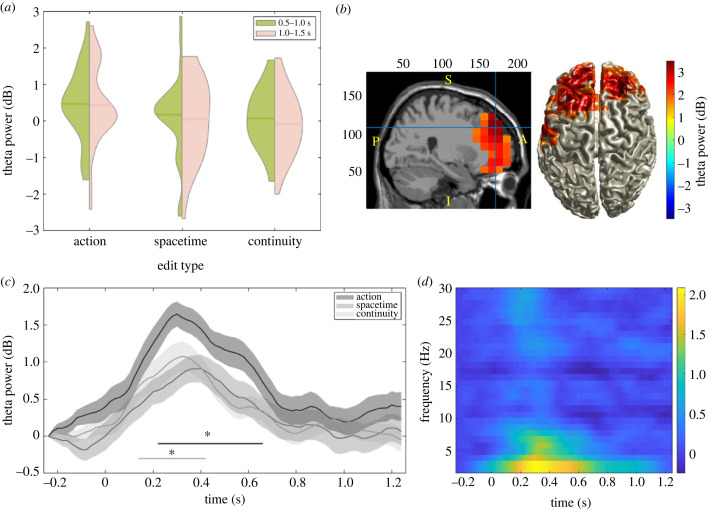
(*a*) Power analysis results: theta power (5–7 Hz, in dB) for each type of edits in the first (green) and second (pink) pre-registered time windows of interest. Solid lines correspond to the group mean of each of the conditions. Filled areas correspond to the distribution of individual data. Both time windows are plotted next to each other for illustrative purposes but it should be noted that no comparison was performed between time windows. Violins made with Bechtold [93]. (*b*) Brain topography of source localised theta activity (5–7 Hz) in dB in the pre-registered time window of interest (0.5–1 s following edits) for all edit types combined. Theta power modulation presents a source consistent with fronto-medial areas. Mask represents the resulting cluster following cluster correction. (*c*) Time-resolved analyses: time courses of fm-theta power (dB) throughout the pre-registered trial length for action (dark grey), space–time (grey) and continuity (light grey) edits averaged across all trials of all participants. The activity has been measured in steps of 20 ms and corrected with respect to the baseline. Significant differences between action and space–time edits and action and continuity edits (*α* < 0.05) are demarked with grey and black horizontal lines. Shaded areas around the activity of theta power indicate the standard error of the mean (s.e.m.). (*d*) Time frequency representation of activity (in dB) at fronto-central electrodes (FCz, Cz, Fz) throughout the pre-registered trial length for all edit types combined.

We then addressed theta power for each edit type separately. In the first time window theta power increased following action edits with respect to baseline (*t*_28_ = 2.485, *p* = 0.01; Cohen's *d* = 0.46), but no significant increase in theta power following space–time edits (*t*_28_ = 0.826, *p* = 0.21), nor continuity edits (*t*_28_ = 0.442, *p* = 0.33) was detected. The results in the second time window were similar: theta power increased significantly following action edits (*t*_28_ = 2.093, *p* = 0.02; Cohen's *d* = 0.39), but not following space–time edits (*t*_28_ = 0.221, *p* = 0.41), nor continuity edits (*t*_28_ = −0.446, *p* = 0.67) (figure 2*a*).

We then compared baseline-corrected post-edit fm-theta power between edit types. All contrasts were one-tailed (as per the directional hypothesis), with *α*-level = 0.05. In the first time window, theta power following action edits was larger than in the other two types of edits, but it was significantly so only when compared to continuity edits (*t*_28_ = 1.725, *p* = 0.048; Cohen's *d* = 0.32), but not for space–time edits (*t*_28_ = 0.972, *p* = 0.17). The second time window showed a similar pattern, as theta power after action edits was significantly larger than continuity edits (*t*_28_ = 2.085, *p* = 0.02; Cohen's *d* = 0.39), and only a trend when compared to space–time edits (*t*_28_ = 1.542, *p* = 0.067). All in all, the results in both time windows show that action edits produced the larger theta increases, and continuity edits the smaller ones.

#### (ii) Exploratory analyses on spatial, temporal and spectral specificity

To ascertain the temporal, spatial and spectral specificity of the effects we performed two exploratory analyses. First, due to the intricate visual nature of films, visual areas and sensorimotor cortex [94] could produce significant neural activity during film viewing. To make sure the theta modulation observed above was consistent with fronto-medial areas specified in our hypothesis, we performed source-localisation using DICS [87]. The cluster-corrected source topography showed a clear source within fronto-medial brain areas, hence consistent with the predicted ACC generator (figure 2*b*).

Second, we estimated the temporal evolution of fm-theta activity surrounding edit boundaries. After selecting the epoch (−0.5 s–1.5 s) and region of interest (electrodes cluster Fz, FCz and Cz), a cluster-based permutation test returned that theta power was greater for action edits than continuity edits from 0.22 s–0.66 s following the edit, and greater than space–time edits from 0.14 s–0.42 s following the edit (figure 2*c*).

Finally, we opened the scope of our analysis over mid-frontal electrodes to other frequency bands in a time-frequency plot. The results (figure 2*d*) show that the contrast within the electrode cluster of interest led to differences mainly in the theta band.

#### (iii) Parieto-occipital alpha oscillations

**Figure 3.**
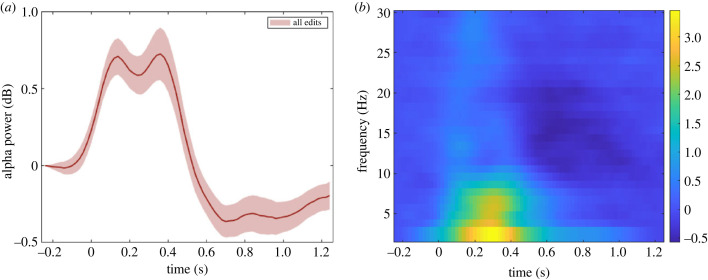
(*a*) Time course of parieto-occipital alpha power activity (8–12 Hz) in dB. (*b*) Time-frequency representation of activity (in dB) at parieto-occipital electrodes throughout the pre-registered trial length for all edit types combined.

As mentioned above, film edits have previously been shown to produce alpha decrements (within 500 ms) at occipital locations [80]. Although this was observed in the case of television, we wanted to see if we observed a similar pattern for parieto-occipital alpha oscillations following film edits (figure 3). Generally, the findings are consistent with this expectation.

#### (iv) Covariations with physical attributes across edits

The real-world nature of cinematic stimuli makes it hard to disentangle the higher-level cognitive processes at play from the many inherent changes in low-level features and particularly salient around editing boundaries [95]. For example, it may be that the pattern of neural activity was affected by one or more physical attributes of the clips. To ascertain that cut characteristics did not influence our results systematically, we computed the correlation between average theta power for each edit and the following stimulus properties: difference in average luminance across shots, difference in average contrast across shots, structural similarity index measures (SSIM) across shots, previous shot duration [95] and difference in sound amplitude across shots (more details can be found in the electronic supplementary material). We found no correlation of theta power with luminance difference across cuts (Spearman's *ρ* = −0.07, *p* = 0.47), nor with contrast difference across cuts (Spearman's *ρ* = −0.07, *p* = 0.47), nor with SSIM index (Spearman's *ρ* = 0.06, *p* = 0.53), nor with previous shot duration (Spearman's *ρ* = 0.15, *p* = 0.13), nor finally with difference of sound amplitude across shots (Spearman's *ρ* = 0.04, *p* = 0.68),^4^ suggesting that power fluctuations around edits were not determined by these physical attributes at editing boundaries.

## 3. Experiment 2: active edit detection task

### (a) Methods

#### (i) Participants and sample size estimation

Forty-two new subjects (mean age: 24.6 years old; 28 female, 14 male) participated in the experiment and were recruited through the Centre for Brain and Cognition of the Universitat Pompeu Fabra participant database. Inclusion criteria comprised no medication. All received 10€/hour in return for their participation, provided written informed consent prior to the study and were naive to the purpose of the experiment. This study was approved by the CIEC ethics board at Parc de Salut Mar (Universitat Pompeu Fabra, Barcelona, Spain).

Sample size was calculated using G*Power [82]. Given our practical affordances and aiming for a statistical power of >0.80 and an effect size of *d* = 0.4 [83], the sample (N) returned by G*Power was of 41.

#### (ii) Materials and procedure

Stimuli comprised the same seven continuous clips as in Experiment 1 (cf. §2a(ii)). The same edits as in Experiment 1 were included in the analysis, as well as the seven shots that were too short to be included in Experiment 1, resulting in *N* = 53 continuity edits, *N* = 31 spatial-temporal discontinuity edits and *N* = 33 action discontinuity edits. This experiment was built using Psychopy's experiment builder (version 2021.2.3, [84]). Participants were seated in a dimly lit and sound-attenuated room (acoustic insulation of about 55 dB). Film clips were displayed on a HP Omen 25 monitor placed at approximately 90 cm from participants' heads, with the soundtrack audible through Logitech loudspeakers. Participants were given the possibility to adjust the seating to a comfortable distance and the sound to a comfortable level (average range of 32–42 dB[A]). Participants were instructed to detect each edit in the clips by right-clicking a mouse once. Edits were defined as a change from one camera shot to another, and participants were shown four examples (two clip excerpts with edits and two without) as well as given the same training task as in Experiment 1 (cf. §2a(iii)), while being monitored for correct performance. As in Experiment 1, following each clip, participants were asked to respond to the same ‘yes/no’ content questions to ascertain attentive watching of films. The list of films that participants had previously seen can be found in the electronic supplementary material.

#### (iii) Exclusion criteria

All participants had more than 70% correct content questions, so none were discarded from further analysis according to this criterion (same threshold as in Experiment 1, cf. §2a(v)), response rates for each clip can be found in the electronic supplementary material. Additionally, in this experiment participants with fewer than 75% correctly detected edits were discarded from further analysis, based on pilot data: firstly our five pilot participants averaged > 97% correctly detected edits and secondly, with 100 random samplings of 20–90% of data, the estimated mean response time (RT) for participants fell within 10% of their actual mean with 70–80% of trials. One participant was discarded based on this criterion and a new participant was recruited (meeting all inclusion criteria) to complete the final sample of *N* = 41.

#### (iv) Statistical analyses

For every participant, RTs more than 2.5 standard deviations away from their mean were discarded (mean = 2.54% responses discarded per participant). Response times faster than 200 ms were not considered for analysis. According to the pre-registered hypothesis, (https://osf.io/s8nwq), RTs to action edits were compared to both other edit types combined (spatial–temporal discontinuity and continuity edits) by means of a one-tailed paired *t*-test, given the directional nature of our hypothesis (RTs should be faster following action edits). An exploratory analysis was conducted by fitting a linear mixed model on R studio (version 2023.06.2+561) to predict RTs with edit type.

Two further exploratory analyses were performed. First, we calculated the proportion of missed edits (edits that were not responded to) and compared it by edit type to address if edit-blindness had occurred [6] using a linear mixed model on R studio (version 2023.06.2+561 [97]). Second, we performed an item analysis, by looking at mean RTs and proportion of missed edits for individual edits. For descriptive purposes, the percentages of each edit type for mean RTs were calculated for each tercile of the overall dataset.

### (b) Results

#### (i) Analysis of mean response times and proportion of missed edits per edit type

We first performed the pre-registered analyses comparing mean RTs following action edits to those following both other edit types combined (spatial–temporal discontinuity and continuity edits) by means of a paired *t*-test. The results showed no significant slowdown (and no significant difference) in RTs (*t*_40_ = 0.027, one-tail, *α* = 0.05; *p* = 0.51). Hereafter we present follow-up and exploratory analyses to better characterise the pattern of results.

First, since the main analysis did not reveal the expected pattern of results when grouping continuity and space–time edits, we decided to unpack these edit types in a new analysis, as in the analyses of Experiment 1. We fitted a linear mixed model to predict (log-transformed) RTs with edit type (R packages lmertest and lme4 [98,99]) including participants as random intercept. We found that edit types played a significant role in determining RTs ($\chi_{2}^{2}=84.2,$ *p*_adj_ < 0.001). *Post-hoc* Bonferroni-corrected tests revealed significantly faster RTs to action edits than to continuity edits (*t* = 2.9; *p*_adj_ = 0.011), significantly faster RTs to space–time edits than to action edits (*t* = 5.82; *p*_adj_ < 0.001), and significantly faster RTs to space–time edits than to continuity edits (*t* = 9.1; *p*_adj_ < 0.001). Mean and standard deviations for RTs and missed edits across edit types are summarised in table 1. All in all, while continuity edits were the slowest to detect as we expected, responses to space–time edits were faster than to action edits, inconsistent with our predictions.

**Table 1.** Mean RTs to edits by edit type and proportion of missed edits by edit type (standard deviations in parentheses).

edit type	response time (ms)				missed edits (%)			
	**all**	action	space-time	continuity	**all**	action	space-time	continuity
mean (SD)	**683 (138)**	685 (154)	632 (132)	721 (162)	**6.5 (3.7)**	1.4 (2.1)	2 (3.3)	12.9 (7.5)

We fitted a linear mixed model to predict edit blindness [6] with edit type (R packages lmertest and lme4 [98,99]), including participants as random intercept. If edit blindness occurred, we expected it to be more prevalent in continuity edits than the other two types. We found that edit blindness was significantly affected by edit type ($\chi_{2}^{2}=180.69,$ *p*_adj_ < 0.001) and happened significantly more often for continuity edits than for action edits (*z* = 10.02; *p*_adj_ < 0.001) and for space–time edits (*z* = 9.8; *p*_adj_ < 0.001).

**Figure 4.**
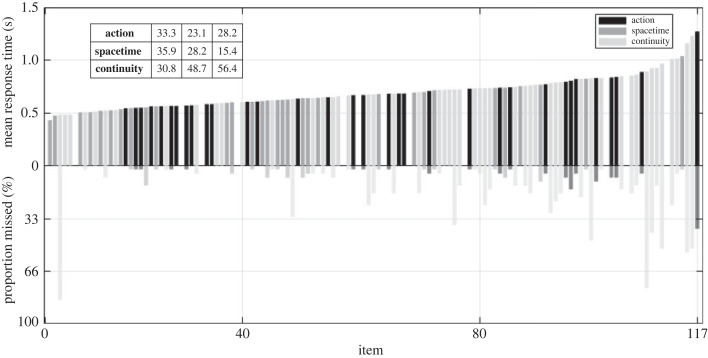
Item analysis. Each bar represents a specific edit in the experiment, with edits ordered along the *x*-axis by mean RT. Above the *x*-axis are the mean RTs (averaged across participants) to each edit, colour coded by edit-type (action, dark grey; space–time, grey; continuity, light grey). Below the *x*-axis is the mean proportion of edit-blindness (averaged across participants) that occurred for each corresponding edit, following the same colour code. Inset table shows the percentage of each edit type falling in each tercile of the distribution represented in the top plot for RTs.

An item analysis was performed by looking at mean RTs and proportion of missed edits for individual edits. Results, along with descriptive percentages of the occurrence of each edit type across each tercile of the data for mean RTs, can be seen in figure 4.

#### (ii) Covariations with physical attributes across edits

For the same reasons as in Experiment 1 (cf. §2b(iv)), we calculated the correlation between mean RTs and physical attributes of editing boundaries (more details can be found in the electronic supplementary material). We found no correlation of mean RTs with luminance difference across cuts (Spearman's *ρ* = 0.11, *p* = 0.24), nor with contrast difference across cuts (Spearman's *ρ* = 0.11, *p* = 0.22), nor with previous shot duration (Spearman's *ρ* = 0.11, *p* = 0.25), nor finally with difference of sound amplitude across shots (Spearman's *ρ* = 0.11, *p* = 0.22), suggesting that RTs to edits were not determined by these physical attributes at editing boundaries. We did find a positive correlation between mean RTs and SSIM index (Spearman's *ρ* = 0.19, *p* = 0.03). While these results could affect the interpretation of our findings, they represent only one of many (and uncorrected) tests, the remainder of which did not yield significant results. Furthermore, they are hardly surprising: since continuity edits only involve a shift in camera position, we expect them to present greater feature overlap (and hence, higher similarity) than those that involve more complex changes across editing boundaries. A Wilcoxon signed rank test revealed that indeed, SSIM values were greater for continuity edits than for action edits (*z* = 2.35, *p* = 0.009). Narrative in film is inextricably linked to visual features by the very design of continuity editing: filmmakers purposefully play with visual features to render edits narratively and visually inconspicuous/salient. Our experimental design does not enable us to disentangle the contribution of visual features to RTs from that of narrative features. We encourage future studies to use original film content to manipulate either narrative or visual features while maintaining the other constant, but it should be noted that any attempt at completely dissociating narrative and visual features of films may compromise their ecological validity.

## 4. Discussion

### (a) Overview of results and limitations

We examined the effects of film edits spanning gaps across different narrative depths by measuring neural responses during passive film viewing and behavioural responses during an explicit edit detection task. The goal was to test the potential role of predictive processes during film watching. At the neural level, we found the predicted power increase in the theta band following edits that supposedly triggered greater surprise signals with respect to those that triggered less surprise, based on levels of narrative depth and intended fluency/disfluency across edits. The involvement of fm-theta was greater surrounding action edits, which represent the greatest narrative shift, in line with the *lingua franca* theory by which it responds to a variety of scenarios involving uncertainty, including novelty [69]. While we were not sure of the latency of this theta effect due to the high-level nature of the stimuli, peak theta activity for all edits, according to the time courses, occurred within the first 0.5 s following edits, with the significant difference between edit types also occurring within (or extending only shortly beyond) this latency, much like other effects observed for fm-theta [27,70], (see §2a(iv) for an explanation of our chosen time windows of analyses). Since these increases were observed specifically for the theta band and varied by edit type, they are likely attributable to a difference in detection and/or resolution of discrepancies, once changes in low-level visual features have been detected. We assume that differential adjustments were required for each edit type based on the organisation of the narrative. Conflict, or error-related fm-theta power includes at least two types of dissociable response: detection and resolution. We expect these to be conflated in the signal of time-frequency EEG analysis [100] and accordingly, are unable to disentangle them in our data nor make any specific interpretations about their respective contribution.

At the behavioural level, the same events discussed above were only partly reflected in the pattern of detection speeds when contrasting action edits with the other types. Instead, RTs to space–time edits, which would in principle constitute a mid-level breach in terms of narrative structure, were significantly faster on average than those to continuity edits (shallow narrative jump) and, more surprisingly, also faster than those to action edits (deep narrative jump). Upon further examination of the data, some results did however reveal an expected pattern. First, mean RTs to action edits were significantly faster than those to continuity edits, and second, continuity edits were missed significantly more often than both action and space–time edits.

Faced with these ambivalent results, the first possibility to consider is that our edit taxonomy was not adequately representative of the difference between our selected edits. Our edit selection was made with a certain number of limitations which may have been too constrictive to ensure a representative range of edits across all edit types of interest (see electronic supplementary material). One difficulty is that the discontinuity physically present in edits and that intended by the filmmaker to be perceived by the viewer do not always overlap. Furthermore, the neurocinematics literature contains many different taxonomies, with varying degrees of precision and containing varying numbers of factors, devices and editing techniques that may contribute to generating predictions [72,101]. Future studies could use more fine-grained taxonomies. Another possibility is that changes in space and time are indeed more salient to the viewer than those in action for reasons different to narrative mismatch, which would explain why the EEG pattern obtained with the same movie fragments confirmed our predictions. However, we have no *a priori* reason to believe this, nor was this pattern reflected in the proportions of missed edits and nor were SSIM values significantly different across action and space–time edits.

Passive viewing, used in Experiment 1, is phenomenologically and physiologically distinct from the active report of stimulus features used in Experiment 2, as well as more representative of movie watching in natural circumstances. Despite using two tasks (with and without report) and encouraging participants to attend to film features for subsequent content questions, we could not eradicate the explicit attention bias towards edits that our behavioural task imposed in Experiment 2. While we are confident that any relative difference in detecting edits of different types should overcome this bias, this limitation in design should be acknowledged as artificially constraining spontaneous detection processes. Finally, the films chosen in our study contained shots and edits that were highly stylised and often technically complex. More stimulus diversity is needed to generalise our findings to other film genres and styles [96].

### (b) Future directions for PP and film editing

While, to the best of our knowledge, the PP framework has not yet been explicitly applied to film editing, we contend that editing boundaries provide excellent opportunities to create manipulable degrees of ‘resolvable’ errors, depending on the depth of narrative gap they span. Film and narrative theorists intuitively understand that a moderate degree of cognitive challenge in film lends itself to aesthetic appeal, so long as it falls within ‘the right’ proportions [102,103] and can be integrated within a broader narrative [104]. Editing provides filmmakers with an unlimited set of tools to creatively induce varying levels of prediction errors at cut boundaries. Another reason why films are valuable stimuli for the PP framework stems from their temporal aspect, which allows for the testing of error dynamics within different levels of narrative organisation as they unfold through the film duration. The topic of cognitive challenge in film has recently been the subject of a collection of works (to which we refer any reader with an interest in the topic) which raise many potentially interesting questions to be addressed with the PP framework [105]. For instance, if error dynamics are central to aesthetic appeal, how does one's overall enjoyment of a film vary with different ratios of mild (resolvable) and intense (unresolvable) discrepancies? What are the parameters of film that most engage predictive processes across shots (average shot length, montage, soundtrack, *mise-en-scène*)? Future studies should attempt to address these questions empirically, as well as test them with an emphasis on editing techniques, on top of film enjoyment at large. Since films are usually viewed in conditions that differ significantly from laboratory settings, we also encourage the combination of active tasks (detection tasks, segmentation tasks, or any task that involve endogenously paying attention to film features) with more ecological passive viewing tasks combined with physiological measures. Regrettably, our EDA data does not allow us to draw conclusions about participants' affective responses to the edits in this respect and future studies could perhaps provide more information on this matter by including reports of enjoyment of different degrees of discrepancies at edit boundaries specifically and by assessing the general enjoyment of films based on the overall cognitive challenge they suppose. Finally, future experiments should also address the contributions of individual differences to the above points since, while the PP theory provides a relevant framework for understanding the relationship between perceptual processes and narrative understanding, this does not entail that it is the sole mechanism involved in what is undeniably an enormously complex and multifaceted process. Given the role of individual experience in determining endogenous predictions (hence, prediction errors) this variability is likely highly relevant (for instance, prior knowledge of the filmic language, etc.).

### (c) Conflict monitoring and predictive processing: a call for a unification of both frameworks

We have argued that narrative understanding in film relies on predictive processes, and tentatively suggested that conflict detection, monitoring and resolution mechanisms could be a plausible vehicle for prediction-error regulation, on account of theoretical and empirical overlaps between both frameworks. This overlap is considerable in the literature, and we call for a thorough assessment of points of convergence between both as well as their respective contribution to an understanding of cognitive function. Although they have not often been considered together, both theories converge on the premise that in everyday life, powerful heuristics minimize prediction errors (or conflict signals) at the service of efficient processing or ‘ecological rationality’ [13,106].

However, many questions remain before these frameworks can truly be considered in common. In §1b we refer to a range of evidence for conflict monitoring at various levels of information processing: however, the conflict literature remains overwhelmingly concerned with stimulus-response conflict. We encourage an in-depth review of the literature to better understand the reason for this imbalance. A consequence of this relative exclusion of perceptual processes from the CMT is an underdevelopment of the hierarchical aspect of the conflict framework compared to that of PP. We believe that what is known of conflict across levels of information processing joins low- and high-precision prediction errors (reflected across different levels of processing), with only the higher-level conflicts reaching awareness, while other occurrences are resolved before.^5^ More research is needed to understand conflict at different levels of processing, how these levels relate to each other, how they trigger the need for differing degrees of attentional adjustments and what determines whether they reach awareness. Another point for future research concerns dissociable signals between monitoring, detection and resolution of error/conflict signal: while we have highlighted overlap between error and conflict signals, how the inferential element of PP relates to the resolution of conflict according to the theory, particularly the mechanisms behind attentional orienting in the dlPFC, remain to be disentangled.

Another point of convergence between both frameworks concerns the role played by affect. In line with recent accounts of the affective value in PP, recent formulations of the conflict framework and models of ACC function characterise conflict as the transition from a negative state (detected conflict) to a positive state (resolved conflict) [19,109], which can result in unexpected reward [19] such as the reward of resolving detected conflict, perhaps leading to information gain [42]. Such a proposal constitutes a radical departure from the longstanding belief that the inherently aversive nature of conflict signals serve as a sensible motivation for their future avoidance and resolution [110,111] and a step towards a more nuanced understanding of affective responses to conflict. Of course, more empirical and theoretical studies should aim to address this point explicitly, consolidating the role of positive affect in conflict mechanisms [112].

Finally, while we have reviewed promising evidence that both frameworks may share common neural substrates, we call for future studies to address these explicitly: in particular, we encourage future studies on PP to include trials of known conflict effects at other levels of information processing (such as between selection of alternative actions in the Stroop task) for subsequent source localisation of common neural activity between both levels. Future studies should also address whether known conflict correlates are engaged during classic PP protocols (for instance known event-related potentials such as auditory or visual mismatch negativity [113,114]). In sum, more research, both theoretical and empirical is needed to understand the exact relationship between both frameworks and their respective contributions to an understanding of perceptual and cognitive function at large.

To sum up, the present study contributes an empirical take on the idea that predictive processes can help foster perceptual integration, in this case by enabling understanding across multiple narrative levels, with the magnitude of low-level conflict signals helping to establish the relative importance of shots within an overarching narrative where different units are hierarchically organised. We hope to have shown that cinema, and editing techniques more specifically, provide an ideal playground to explore this perceptual feat.

## 5. Conclusion

While film fragments are more complex than classical laboratory-bound stimuli, filmmakers will disagree with neuroscientists that they represent a step towards greater ecological validity, given the vast amount of editing and shaping filmic sequences require. However, although the success of a film depends largely on montage techniques, these are nothing if not received by a flexibly adaptive perceptual system able to make sense of narrative continuity across cuts. Addressing these processes not only helps understand film viewing, but also reveals important aspects of perceptual function, not in spite of but due to its very difference from the flow of everyday sensory experience. We have argued that, in film, editing boundaries are the central tool by which filmmakers lead viewers to make predictions about their visual experience, be it to sustain their interest, create engagement or immerse them within the narrative. We provide some initial evidence that these dynamics can be captured by mechanisms consistent with predictive processes, via conflict and error signals at the neural and behavioural levels. We found that fm-theta oscillations reflect jumps across different degrees of narrative depth, with a larger response to the most profound narrative shifts as opposed to those supposedly made inconspicuous by film theory. This continues a line of research associating fm-theta power with responses to novelty, conflict and error signals [69] during perceptual inference in various domains. This pattern was also reflected behaviourally with certain edits being more or less salient to the viewers, which we attribute to their differing degrees of predictability and thus, differing need for the invocation of adjustments. We believe our contribution lays some groundwork for the study of cinema from the viewpoint of prediction errors and conflict signals, in an attempt at broadening the understanding of the role these neural signals play in perceptual processing more generally and their relationship to each other.

## Data Availability

Relevant data and analysis scripts can be accessed from OSF repository: https://osf.io/98d3p/ [115]. Please note that due to storage limitations, only half of the electrodermal activity dataset could be made accessible; the other half is available upon request. Supplementary material is available online [116].

## Endnotes

1 This was once suggested to be the only type of conflict the ACC responds to [21].

2 We use the term ‘uncertainty’ here to encompass prediction error, challenge, conflict, novelty, surprise and other related states.

3 With the exception of *Le Ballon Rouge*, which contains three times a vocal interjection in French, with subtitles.

4 Please note that the *p*-values reported here and in §3b(ii) are not corrected for multiple comparisons.

5 It is worth pointing out that binocular rivalry has been studied both from the perspective of PP and conflict monitoring [29,107], as is the case with the McGurk illusion [27,108]. In both cases, one could at least make the assumption that conflict occurs and is resolved below the threshold of awareness.
